# Digital behaviour change interventions to promote physical activity in overweight and obese adolescents: a systematic review protocol

**DOI:** 10.1186/s13643-022-02060-w

**Published:** 2022-09-05

**Authors:** Puteri Shanaz Jahn Kassim, Noor Azimah Muhammad, Nur Faraheen Abdul Rahman, Sherina Mohd Sidik, Cecilia A. Essau, Shamsul Azhar Shah

**Affiliations:** 1 Department of Family Medicine, Faculty of Medicine and Health Sciences, Universiti Putra Malaysia, 43400 Serdang, Selangor Malaysia; 2 Department of Family Medicine, Faculty of Medicine, Universiti Kebangsaan Malaysia, Cheras, 56000 Kuala Lumpur, Malaysia; 3 Research Department of Primary Care and Population Health, Institute of Epidemiology and Health Care, University College London, London, WC1E 7HB UK; 4 Department of Primary Health Care and Medical Education Unit, Faculty of Medicine and Health Sciences, Universiti Sains Islam Malaysia, 71800 Nilai, Negeri, Sembilan Malaysia; 5 Department of Psychiatry, Faculty of Medicine and Health Sciences, Universiti Putra Malaysia, 43400 Serdang, Selangor Malaysia; 6 Department of Psychology, Centre for Research in Psychogical Wellbeing, University of Roehampton, London, SW15 5PJ UK; 7 Department of Community Health, Faculty of Medicine, Universiti Kebangsaan Malaysia, Cheras, 56000 Kuala Lumpur, Malaysia

**Keywords:** Digital intervention, Behaviour change, Digital behaviour change, Behavioural interventions, Adolescent, Physical activity, Obesity, Systematic review protocol

## Abstract

**Background:**

With the increasing prevalence of obesity in youth, behavioural interventions to alter its modifiable risk factors such as physical activity can support the management of this epidemic. Digital behaviour changes interventions (DBCI) such as mobile applications, websites and wearables have the potential to reach many adolescents to promote physical activity as its use may be more accessible, effective and engaging compared to traditional face-to-face approaches. However, there is insufficient evidence on their use at promoting physical activity amongst overweight and obese adolescents. This review aims to assess the effectiveness of DBCIs aiming to increase physical activity in overweight and obese adolescents (aged 10–19 years) and the behaviour change techniques used in these interventions.

**Methods:**

Electronic databases (MEDLINE, EMBASE, PsycINFO, CINAHL, Cochrane and Scopus) will be searched for English language studies from January 2000 to December 2022 using appropriate search terms relating to digital interventions, physical activity, adolescents and obesity. Experimental studies (either randomised or non-randomised controlled trials) assessing effects of DBCIs on physical activity behaviour, objectively or subjectively measured, in overweight and obese (body mass index [BMI] ≥ 85th percentile for age) adolescents will be eligible for inclusion. Intervention characteristics will be coded using the Template for Intervention Description and Replication (TIDieR) checklist and the BCT taxonomy v1. Risk of bias and the overall quality of the included studies will be assessed using Cochrane’s Collaboration’s tool and GRADE approach respectively. If the data allows, meta-analyses using random effect models will be conducted to assess the effects of DBCIs on physical activity.

**Discussion:**

The proposed systematic review will summarise the effectiveness of digital behaviour change interventions aiming to increase physical activity in overweight and obese adolescents, as well as adding new information on the behaviour change techniques used in these interventions. The findings of this review will facilitate stakeholders with a current, rigorous and reliable research base to support the development and implementation of effective health promotion interventions for this population.

**Systematic review registration:**

PROSPERO CRD42021270008.

**Supplementary Information:**

The online version contains supplementary material available at 10.1186/s13643-022-02060-w.

## Background

Obesity is a significant public health problem that affect adolescents today. The World Health Organisation has cited overweight and obesity, as well as physical inactivity as two out of five leading global risk factors for mortality [1]. Around 80% of obese adolescents will remain obese in adulthood [2]. Compared to non-overweight adolescents, becoming overweight predisposes youth to adverse health effects, such as hypertension, type 2 diabetes, obstructive sleep apnoea, aggravated asthma symptoms and a reduced life span [3].

One of the major factors contributing to the obesity epidemic is physical inactivity [4]. Even though physical activity has been linked to important health benefits such as prevention of several chronic medical conditions [5], a large number of adolescents today do not meet the recommended guidelines of at least 60 min of moderate-to-vigorous physical activity (MVPA) daily [6], which in turn leads to the increasing prevalence of obesity in this population [7]. Overweight and obese adolescents, in particular, has been shown to engage in less physical activity than adolescents who are of healthier weight [8]. This population perceives several barriers towards engaging in physical activity at the individual, interpersonal and environmental level, potentially leading to negative self-perception [9]. Low levels of actual and perceived physical competence amongst overweight and obese youth may inversely influence their motivation to participate or remain consistent in physical activity, which deprives them of further opportunities to improve their skill and competency in physical activity [10]. Furthermore, being overweight or obese predisposes adolescents to a greater degree of victimisation related to weight stigma, leading to difficulty in forming relationships with peers, which may affect their participation in physical activity [9, 11].

As physical activity plays crucial role in modifying risk of obesity and onset of other non-communicable diseases, it serves as important target for risks prevention strategies and health promotion interventions. Furthermore, it is particularly important for overweight and obese adolescents to have increased physical activity, which brings health benefits by not only contributing to weight loss, but also to physical and mental health benefits such as enhanced self-esteem and cognitive function [12].

Digital behaviour change interventions (DBCIs) utilise digital technologies that encourage and support behaviour change to improve and maintain health, through prevention and management of health problems [13]. Examples of DBCIs include smartphone applications, website, SMS text messaging programmes, email, and body and environmental sensors. DBCIs are utilised for health promotion by providing individual support in the “real world” to change specific behaviours in specific contexts [14]. Previous systematic reviews have investigated the evidence on the effectiveness of digital interventions to improve diet quality and increase physical activity in adolescents in general [15] as well as weight-related outcomes [16]. However, the data on effects of DBCI targeting overweight and obese adolescents on physical activity behaviour are still lacking [16].

Various strategies and behaviour change techniques (BCTs) are used in behaviour change interventions to facilitate participants’ self-regulation abilities during behaviour change [17]. BCT is referred as the irreducible “active ingredient” or intervention component that is fundamental to behaviour change [18]. Specifying the active components of an intervention is important to enable implementation and replication of effective intervention [18]. The Behaviour Change Techniques Taxonomy v1 (BCTTv1) is a reliable, comprehensive, and theory-based taxonomy which contains 93 hierarchically clustered BCTs, distributed in 16 groups, that allows systematic description, evaluation, and replication of the “active ingredients” of interventions [18]. “Goal-setting”, “problem solving” and “self-monitoring” are examples of common BCTs used in behaviour change interventions [15, 19, 20]. Based on a review of internet-based interventions for health promotion, theory-based intervention and interventions incorporating more BCTs have shown to have greater impacts in the general population [21]. Previous reviews that examined the effectiveness of digital interventions in promoting physical activity behaviour has mainly targeted adolescents within the normal weight range, and did not identify the BCTs using the BCT Taxonomy v1 [15, 22]. Therefore, this review aims to fill in this gap by examining the behaviour change techniques using the 93 item BCT taxonomy v1 [18] used in digital physical activity interventions targeting overweight and obese adolescents.

The growing interest of DBCIs, along with a rising prevalence of obesity and physical inactivity in adolescents necessitates an update of evidence in this area by examining the effects of these technology-based interventions on physical activity behaviour in overweight and obese adolescents. The findings from this review will add to the literature as evidence base in guiding the development of effective behaviour change interventions in this population. Accordingly, this systematic review aims to examine whether DBCIs targeting an increase in physical activity are beneficial for overweight and obese adolescents. Specifically, we set out to investigate the following research questions:

1. Are digital behaviour change interventions (DBCI) effective at promoting a change in physical activity behaviour when comparing the intervention with control groups amongst overweight and obese adolescents?
2. Which behaviour change techniques (BCTs) were used in these interventions?

## Methods

The protocol was prepared following the Preferred Reporting Items for Systematic Reviews and Meta-Analysis Protocols (PRISMA-P) 2015 statement [23] (Additional file 1). The final review will be reported using the Preferred Reporting Items for Systematic Review and Meta-Analysis (PRISMA) statement [24].

### Study registration

This systematic review protocol is registered in the PROSPERO international prospective register of systematic reviews (registration number: CRD42021270008; https://www.crd.york.ac.uk/PROSPERO/).

### Eligibility criteria

#### Type of studies

Randomised controlled trials (RCTs), either parallel group or cluster-randomised, and non-randomised controlled trials (i.e. quasi-experimental studies) will be included in the review.

The included studies will be identified based on the population, intervention, comparator, and outcome (PICO) framework.

#### Type of participants

The population of interest are participants within the adolescent age range, which is defined as people aged 10–19 years [25]; who are either overweight or obese, following the CDC criteria (BMI > 85th centile) [26] or WHO growth reference (greater than 1 standard deviation above the WHO Growth Reference median) [27].

#### Type of interventions

Interventions will be included if it incorporates any form of digital intervention, including internet-based, web-based, mobile/apps, social media platforms, text messaging, wearables, smartphones or body and environmental sensors such as exergaming, that aims to increase physical activity. Interventions which incorporates non-digital components will be included only if the digital component is the primary element of the intervention.

#### Type of comparators

Comparators can either be usual care, a wait-list comparator or comparison to a non-digital intervention. However, as the focus of the review is effectiveness of DBCI, studies will only be included if the comparator groups did not receive any digital or technology-based interventions.

#### Type of outcome measures

The primary outcome of interest is physical activity, either measured objectively (i.e. accelerometer or pedometer) or subjectively (e.g. self-report) at baseline and post-intervention. Changes in physical activity can be expressed as minutes per day/week of moderate to vigorous physical activity (MVPA); METs per week (METs/week); changes in daily steps, or percentage of participants meeting recommendations for PA. A hierarchy of preferred metrics for PA outcome is as follows: (1) minutes of MVPA, (2) total PA minutes, (3) daily step counts and (4) daily energy expenditure. If more than one relevant outcome is reported within a study, a hierarchy of preferred metrics for PA outcome will be extracted favouring MVPA followed by daily step counts and energy expenditure. If no objective measure is available, we will use self-reported measures. PA could be the sole target outcome of an intervention, or one of the target behaviours within an intervention aiming to change multiple health behaviours.

### Exclusion criteria

Studies will be excluded if (i) the participants were not exclusively within the adolescent age range, (ii) if it targeted adolescents with normal weight status, (iii) if it did not report physical activity outcome and (iv) if the intervention is not largely delivered via digital or technology-based platform or if the participants were not directly utilising the DBCI.

### Adverse outcomes

All reported adverse effects will also be extracted where available (i.e. worsened quality of life, injuries).

### Information sources and search strategy

Relevant studies published in English from January 2000 to December 2022 will be identified using six databases (MEDLINE, PsycINFO, Embase, CINAHL, Scopus, Cochrane Library). The year 2000 is selected based on a prior bibliometric analysis that showed that eHealth research targeting physical activity was almost exclusively published from this year onwards [28]. Search terms and Medical Subject Heading (MeSH) will include all possible terms relating to adolescents, physical activity, obesity and digital intervention (Additional File 2). Additional studies via searches of the reference lists of included articles will also be screened.

### Screening procedure and data management

The first author (PS) will perform the initial search. Results will be downloaded into a referencing software (EndNote X9, Clarivate Analytics, Philadelphia, PA, USA) to remove duplicates. The remaining results will then be transferred to Covidence® where additional duplicates will be removed. Titles and abstracts of the retrieved studies will be screened independently by PS and NF to exclude irrelevant studies. Full texts will be obtained for relevant studies and reviewed by PS and NF to determine whether they fulfil the inclusion criteria. Any discrepancies will be resolved through discussion between the PS and NF. If a decision could not be reached, a third senior reviewer (NAM) served as an adjudicator to reach final eligibility decision.

### Data extraction

Two authors (PS and NF) will independently extract the relevant population and intervention characteristics using a pre-tested standard data extraction form for the selected studies that fulfil the inclusion criteria. Any disagreements will be resolved by discussion, or if required, by a third reviewer (NAM). The outcomes to be extracted include: publication details (author, year, country of origin) setting, study characteristics (which include aims of the study, study design, sample size, study population demographics and baseline characteristics), recruitment strategy; inclusion criteria, details of the DBCI (including duration) and control group conditions which will be described according to the TIDieR (Template for Intervention Description and Replication) checklist [29]; description of outcome measures (objective measurement or self-report) including sub-category of PA (e.g. minutes of MVPA, step counts); effects on physical activity and any additional outcomes analysed, if reported (e.g. anthropometric, dietary, mental, or social measures, quality of life). In addition, the values of intraclass correlation coefficient (ICC), the number of clusters and the cluster sizes for clustered studies will also be extracted. If there are multiple publications reporting the same study, all of the available data will be extracted and integrated, where possible. When required, any unclear or missing information of the study will be sought from the authors of the articles for clarification and additional results. Characteristics of interventions and participants will be summarised in tables.

### Coding of BCTs

Specific behaviour change techniques (BCT) used in the DCBI will be coded independently by two reviewers (PS and NF) according to the Behaviour Change Techniques Taxonomy v1 (BCTTv1) [18]. They will be checked for accuracy by a third reviewer (CE). A BCT will only be coded if its use was clearly described in the intervention. If studies included multiple intervention arms, the BCTs and outcome data for each intervention arm versus the control condition will be extracted.

### Risk of bias assessment

Assessment of risk of bias for included studies will be conducted according to the criteria outlined in the Cochrane Handbook for Systematic Reviews of Interventions [30]. Specifically, the randomised trials will be examined for bias using the version 2 of the Cochrane Risk of bias tool (RoB2), which assesses several key areas of potential bias: randomisation process; deviations from intended interventions; missing data; measurement of outcome and selection of the reported result [30]. An overall risk of bias judgement will be assigned to each trial based on: low risk of bias if low risk of bias for all domains; some concerns if at least one domain has some concerns but not domains judged to be at high risk of bias and high risk of bias if high risk of bias in any domains or some concerns in multiple domains. The RoB2 tool will also be used to assess the risk of bias of cluster-randomised trials, which will address the potential bias in the following domains: randomisation process; timing of identification and recruitment of participants; deviations from intended interventions; missing outcome data; measurement of the outcome and selection of the reported result [31]. Quasi-experimental and non-randomised trials will be assessed using the Risk of Bias tool for Nonrandomised Studies of Interventions (ROBINS-I) tool, which will assess risk of bias due to confounding; selection bias; classification of interventions; deviations from intended interventions; missing data; measurement of outcomes and selection of the reported result. Each study will be assigned an overall risk of bias judgement (low, moderate, serious, and critical risk) [32]. Two reviewers (PS and NF) will independently assess the risk of bias for each study. Any disagreements will be resolved with a consensus or consultation with a third reviewer (NAM), where necessary.

### Quality of evidence

The quality of the evidence for primary outcomes will be evaluated using The Grading of Recommendations Assessment, Development and Evaluation (GRADE) guidelines [33, 34]. Assessment will be performed in the following domains: risk of bias, consistency of effect, imprecision, indirectness and publication bias. The presence of publication bias will be detected by funnel plots and Egger’s regression test using RevMan software, if there are 10 or more studies in the meta-analysis [35]. Based on the GRADE guidelines, the quality of the studies will be judged as ‘high’, ‘moderate’, ‘low’ or ‘very low’ depending on our confidence on the accuracy of the effect estimates [33, 34]. Both RCTs and non-randomised studies of interventions (NSRI) will commence as high quality; but they can be downgraded to lower levels of evidence if serious limitations exist in any of the five domains. However, owing to the inherent risk of bias due to lack of randomisation, which include confounding and selection bias, the evidence for NSRI will be downgraded by two levels, if there are serious problems in any one domain [34]. GRADE will be carried out independently by two review authors (PS and NF), and a third senior author (NAM) will mediate if there are any disagreements.

### Data synthesis

#### Effects of DBCI

If sufficient outcome data is available from at least two studies, results from the included trials will be combined in a meta-analysis. For studies utilising both continuous and dichotomous outcome data to be included in the meta-analyses, appropriate statistical methods will be applied for each type of outcome data. For continuous outcome data (e.g. MVPA minutes/week, steps/day), standardised mean differences (SMDs) and standard deviations (SD) with 95% CI will be calculated using between-group changes from baseline values and will be interpreted based on Cohen’s classification as small (0.2–0.5), medium (0.5–0.8) and large (0.8) [36, 37]. As for dichotomous outcomes (i.e. percentage of participants meeting recommendations for PA), risk ratios with 95% CI will be calculated and converted to SMDs using the Chinn 2000 equation [38, 39]. For cluster RCTs, we will examine whether the study authors have used an appropriate method of analysis to account for clustering. For studies which did not account for clustering, intraclass correlation coefficient (ICC) will be used in the meta-analyses to calculate the effective sample size in order to ensure the effect of clustering is taken into account in the analyses, in accordance with the Cochrane Handbook [31]. When interventions included multiple PA intervention arms, the sample size of the control group will be split equally between intervention arms to allow for separate comparisons [39]. Evidence from RCTs and non-RCTs will be synthesised separately. As for RCTs, a meta-analysis will be conducted using RevMan software [40]. Depending on the availability of statistical data, a random-effects meta-analysis model will be used to calculate the standardised study effect sizes as it is anticipated that the included studies will be varied in terms of intervention components, as well as PA assessment and comparator groups (i.e. no intervention, non-digital intervention). The estimates of effects will be weighted by the inverse of variance, giving more weight to larger trials. As for non-RCTs, the following factors will be carefully evaluated prior to deciding to conduct a meta-analysis: (1) study design weakness, (2) study execution following a risk of bias assessment, (3) confounding and selection bias and (4) potential reporting biases. Adjusted effects are preferred for non-randomised trials, if both unadjusted and adjusted intervention effects are reported [41]. In the case that meta-analysis is precluded, we will either summarise the effect estimates or combine *P* values, based on guidelines in the Cochrane Handbook [42]. If the data is not able to be pooled in a meta-analysis, a narrative review will be conducted following Synthesis Without Meta-analysis guidance [43], and summary of findings using tables of studies, participants’ characteristics, details of DBCI and PA outcomes of the included studies.

#### Assessment of heterogeneity and subgroup analyses

Heterogeneity will be evaluated using Higgins *I*^2^ statistics with values above 75% and *p* < 0.10 (as specified by the guidance in the Cochrane Handbook for Systematic Reviews of Interventions) indicating high heterogeneity across studies and considered eligible for subgroup analysis in order to determine the source of the heterogeneity [44]. If the meta-analysis includes 10 or more studies, subgroup analyses will be performed to investigate the essential elements of DBCI in promoting physical activity. The subgroup analyses will include the following: DBCIs that focused solely in promoting PA as opposed to multiple health behaviours (such as dietary component or weight loss) and the duration of intervention (less than 3 months, 3–6 months, 6 months to 1 year, more than 1 year), as well as type of PA measurement (self-report vs objective). Sensitivity analyses will be performed to determine the effects of studies with a high risk of bias on the overall findings with and without these studies. In addition, the sensitivity analysis will also determine the effect of different ICC values which were used to adjust for clustering, which have not been accounted for in original cluster trials.

## Discussion

Physical inactivity has been established as one of the significant risk factors for obesity and other non-communicable diseases such as heart disease, type 2 diabetes and cancer. Even though many technology-based interventions have been implemented to promote physical activity amongst adolescents, the effectiveness of these interventions in changing these behavioural outcomes in overweight and obese adolescents is still unclear. Aiming to fill in this gap, this review will facilitate understanding of the current digital behaviour change interventions, and its impact on physical activity, focusing on overweight and obese adolescents. In addition, the use of behaviour change taxonomy to examine the BCT content of current digital interventions in this review will enable a more thorough and systematic analysis of the potential ‘active ingredients’ of these interventions in facilitating successful behaviour strategies. The strengths, limitations and implications of integration between technology and behaviour change strategies will be valuable to relevant stakeholders in helping to inform and improve the design, development, acceptability and efficacy of digital behaviour change interventions to improve healthy lifestyle behaviour of our future generation.

## Supplementary Information

**Additional file 1.** PRISMA-P Checklist.

**Additional file 2.** Example search strategy for Medline (via PubMed).

## Data Availability

Not applicable.

## Abbreviations

PA: Physical activity
DBCIs: Digital behaviour change interventions
BCT: Behaviour change technique
BCTTv1: Behaviour Change Techniques Taxonomy v1
CDC: Centre of Disease Control
RCT: Randomised Controlled Trail
MVPA: Moderate-to-vigorous physical activity
WHO: World Health Organisation
PRISMA-P: Preferred Reporting Items for Systematic Reviews and Meta-Analysis Protocols
PRISMA: Preferred Reporting Items for Systematic Review and Meta-Analysis
MEDLINE: Medical Literature Analysis and Retrieval System Online
CINAHL: Cumulative Index to Nursing & Allied Health Literature
EMBASE: Excerpta Medica Database
PsycINFO: Psychological Abstracts
Scopus: Elsevier Bibliographic Database
GRADE: Grading of Recommendations Assessment, Development and Evaluation
