# Supplementary material for: Digital behaviour change interventions to promote physical activity in overweight and obese adolescents: a systematic review protocol
Source: Syst Rev. 2022 Sep 5;11:188. doi: 10.1186/s13643-022-02060-w (PMC9446806; doi:10.1186/s13643-022-02060-w)
Supplement: Supplementary file 2 — Additional file 2. Example search strategy for Medline (via PubMed). [file 13643_2022_2060_MOESM2_ESM.docx]

Additional File 2: Sample MEDLINE search strategy (via PubMed)

**Adolescent**

| **Concept** | **Search terms** | **Type of search** | **Results** |
| --- | --- | --- | --- |
| #1 | Adolescent[MeSH] | MeSH terms | 2,124,603 |
| #2 | teen*[Title/Abstract] OR youth[Title/Abstract] OR juvenile[Title/Abstract] | Keyword TI/AB | 186,777 |
| #3 | #1 OR #2 |  | 2,215,394 |

**Obesity**

| **Concept** | **Search terms** | **Type of search** | **Results** |
| --- | --- | --- | --- |
| #4 | Obesity[MeSH] AND Pediatric Obesity[MeSH] | MeSH terms | 10,721 |
| #5 | obese[Title/Abstract] OR obesity[Title/Abstract] OR overweight[Title/Abstract] OR unhealthy weight[Title/Abstract] OR adolescent obesity[Title/Abstract] | Keyword TI/AB | 354,712 |
| #6 | #4 OR #5 |  | 355,538 |

**Physical Activity/ Sedentary Behaviour**

| **Concept** | **Search terms** | **Type of search** | **Results** |
| --- | --- | --- | --- |
| #7 | Exercise[MeSH] | MeSH terms | 217,682 |
| #8 | “Physical Fitness”[MeSH] | MeSH terms | 33,310 |
| #9 | physical activity[Title/Abstract] OR recreation[Title/Abstract] OR workout[Title/Abstract] OR sports[Title/Abstract] OR fitness[Title/Abstract] OR MVPA[Title/Abstract] OR running[Title/Abstract] OR walking[Title/Abstract] OR cycling[Title/Abstract] OR swimming[Title/Abstract] OR gymnastics[Title/Abstract] OR skipping[Title/Abstract] OR dancing[Title/Abstract] | Keyword TI/AB | 481,059 |
| #10 | #7 OR #8 OR #9 |  | 592,689 |
| #11 | Sedentary behavior[MeSH] | MeSH terms | 11,525 |
| #12 | "physical inactivit*"[Title/Abstract] OR sedentary[Title/Abstract] OR "sedentary behaviour"[Title/Abstract] OR sitting[Title/Abstract] OR inactive[Title/Abstract] | Keyword TI/AB | 162,785 |
| #13 | #11 OR #13 |  | 164,953 |
| #14 | #10 OR #13 |  | 717,656 |

**Digital intervention**

| **Concept** | **Search terms** | **Type of search** | **Results** |
| --- | --- | --- | --- |
| #15 | Telemedicine[MeSH] | MeSH terms | 36,855 |
| #16 | “Internet-Based Intervention”[MeSH] | MeSH terms | 704 |
| #17 | “Wearable Electronic Devices”[MeSH] | MeSH terms | 14,796 |
| #18 | website[Title/Abstract] OR web-based[Title/Abstract] OR computer-based[Title/Abstract] OR online[Title/Abstract] OR "digital intervention"[Title/Abstract] OR eHealth[Title/Abstract] OR e-health[Title/Abstract] OR "electronic health"[Title/Abstract] OR "mobile health"[Title/Abstract] OR mHealth[Title/Abstract] OR m-Health[Title/Abstract] OR telehealth[Title/Abstract] OR "health technology"[Title/Abstract] OR "mobile phone"[Title/Abstract] OR smartphone[Title/Abstract] OR "smart phone"[Title/Abstract] OR cellphone[Title/Abstract] OR "cell phone"[Title/Abstract] OR "digital device"[Title/Abstract] OR "digital technology"[Title/Abstract] OR "mobile device"[Title/Abstract] OR "mobile technology"[Title/Abstract] OR "mobile app"[Title/Abstract] OR "mobile application"[Title/Abstract] OR "mobile phone app"[Title/Abstract] OR "mobile phone application"[Title/Abstract] OR "smartphone app"[Title/Abstract] OR "smartphone application"[Title/Abstract] OR "smart phone app"[Title/Abstract] OR "smart phone application"[Title/Abstract] OR "cellphone app"[Title/Abstract] OR "cellphone application"[Title/Abstract] OR "cell phone app"[Title/Abstract] OR "cell phone application"[Title/Abstract] OR "tablet app"[Title/Abstract] OR "tablet application"[Title/Abstract] OR "text messaging"[Title/Abstract] OR SMS[Title/Abstract] OR "short message service"[Title/Abstract] OR email[Title/Abstract] OR e-mail[Title/Abstract] OR "electronic mail"[Title/Abstract] OR wearable[Title/Abstract] OR "wearable tracker"[Title/Abstract] OR "fitness tracker"[Title/Abstract] OR Fitbit[Title/Abstract] OR "fitness watch"[Title/Abstract] OR smartwatch[Title/Abstract] OR "smart watch"[Title/Abstract] OR "activity tracker"[Title/Abstract] OR "activity sensor"[Title/Abstract] OR "activity monitor"[Title/Abstract] OR "movement tracker"[Title/Abstract] OR "movement sensor"[Title/Abstract] OR "movement monitor"[Title/Abstract] OR "self-monitoring device"[Title/Abstract] OR "social media"[Title/Abstract] OR Facebook[Title/Abstract] OR YouTube[Title/Abstract] OR Twitter[Title/Abstract] OR Instagram[Title/Abstract] OR WhatsApp[Title/Abstract] OR TikTok[Title/Abstract] OR blog[Title/Abstract] OR "video games"[Title/Abstract] OR pedometer[Title/Abstract] OR accelerometer[Title/Abstract] | Keyword TI/AB | 365,650 |
| #19 | #15 OR #16 OR #17 OR #18 |  | 396,992 |

**Publication type-Filter**

| **Search name** | **Search filter** | **Results** |
| --- | --- | --- |
| #20 | Randomized Controlled Trial[pt] | 546,146 |
| #21 | Controlled Clinical trial[pt] | 635,693 |
| #22 | Randomized[Title/Abstract] | 582,228 |
| #23 | Trial[Title/Abstract] | 671,956 |
| #24 | Randomly[Title/Abstract] | 367,537 |
| #25 | Placebo[Title/Abstract] | 228,067 |
| #26 | #20 OR #21 OR #22 OR #23 OR #24 OR #25 | 1,496,065 |

## Final search results and summary

| **Search name** | **Search filter** | **Results** |
| --- | --- | --- |
| #27 | #3 AND #6 AND #14 AND #19 | 928 |
| #28 | #3 AND #6 AND #14 AND #19 AND #26 | 238 |
| #29 | #3 AND #6 AND #14 AND #19 AND #26 Filters: English, Adolescent | 202 |
